# Prevalence of Post-traumatic Stress Symptoms and Its Associations With Quality of Life, Demographic and Clinical Characteristics in COVID-19 Survivors During the Post-COVID-19 Era

**DOI:** 10.3389/fpsyt.2021.665507

**Published:** 2021-05-21

**Authors:** Yuan Yuan, Zi-Han Liu, Yan-Jie Zhao, Qinge Zhang, Ling Zhang, Teris Cheung, Todd Jackson, Guo-Qing Jiang, Yu-Tao Xiang

**Affiliations:** ^1^Chongqing Mental Health Center, Chongqing, China; ^2^Unit of Psychiatry, Department of Public Health and Medicinal Administration, Faculty of Health Sciences, University of Macau, Macao SAR, China; ^3^Centre for Cognitive and Brain Sciences, University of Macau, Macao, China; ^4^Institute of Advanced Studies in Humanities and Social Sciences, University of Macau, Macao, China; ^5^The National Clinical Research Center for Mental Disorders, School of Mental Health, Capital Medical University, Beijing, China; ^6^Beijing Key Laboratory of Mental Disorders Beijing Anding Hospital, School of Mental Health, Capital Medical University, Beijing, China; ^7^The Advanced Innovation Center for Human Brain Protection, School of Mental Health, Capital Medical University, Beijing, China; ^8^School of Nursing, Hong Kong Polytechnic University, Hong Kong, China; ^9^Department of Psychology, University of Macau, Macau, China

**Keywords:** PTSS, quality of life, COVID-19 survivors, depressive symptoms, post-COVID-19 era

## Abstract

**Background:** The prevalence of post-traumatic stress symptoms (PTSS) in COVID-19 survivors is unclear. This study examined the prevalence of PTSS and its association with quality of life (QOL) among COVID-19 survivors during the post-COVID-19 era in China.

**Methods:** This was a comparative, cross-sectional study. PTSS, depressive symptoms, and QOL were assessed with standardized instruments.

**Results:** A total of 134 COVID-19 survivors and 214 non-infected controls (healthy controls hereafter) were recruited. Among COVID-19 survivors, the PTSS prevalence was 18.66% (95%CI: 11.98–25.34%), which was significantly higher than that (5.61%, 95%CI: 2.50–8.71%) of healthy controls (*P* < 0.001). After controlling for covariates, an analysis of covariance (ANCOVA) showed that COVID-19 survivors had a higher PTSS total score than did healthy controls [*F*_(1,348)_ = 4.664, *P* = 0.032]. A separate ANCOVA revealed there were no significant differences in overall QOL between COVID-19 survivors with and without PTSS [*F*_(1,348)_ = 1.067, *P* = 0.304]. A multiple logistic regression analysis showed that more severe depressive symptoms were significantly associated with PTSS in COVID-19 survivors (OR = 1.425, *P* < 0.001).

**Conclusions:** PTSS were more severe in COVID-19 survivors compared to healthy controls in the post-COVID-19 era. Considering their negative impact on daily life and functional outcomes, regular assessment and appropriate treatments of PTSS should be conducted in COVID-19 survivors.

## Introduction

Since the coronavirus disease 2019 (COVID-19) was first reported in Wuhan, Hubei province in December 2019, it has been found in over 200 countries and territories as a pandemic. As of the middle of 2020, the COVID-19 epidemic had been well-controlled in China, although imported cases were identified in some areas occasionally (1). The Chinese health authority reported that 100,877 people had been infected by January 2021, of whom, 93,449 (92.64%) had recovered (2). Apart from Hubei province, Chongqing was one of cities most affected in the pandemic. In Chongqing, the first COVID-19 case was found in January 2020. By March, 2021, 576 residents had been infected with COVID-19, of whom, 570 (99%) have recovered. However, previous studies have found that long-term psychological distress, including post-traumatic stress disorder (PTSD), may result from infectious disease outbreaks among survivors (3, 4).

PTSD is a common psychiatric disorder based upon stress experiences following traumatic events such as wars, natural disasters and traffic accidents (5). The disorder is characterized by a host of post-traumatic stress symptoms (PTSS) including intrusive thoughts, vigilance, avoidance of trauma-related stimuli, emotional numbing and physiological hyper-arousal (5). Exposure to infectious disease epidemics could result in psychological trauma and other psychiatric problems including PTSD and PTSS. For instance, previous studies found that more than half of survivors of severe acute respiratory syndrome (SARS) were diagnosed with PTSD in the post-SARS era (6) and 39% (95% CI: 31–47%) of the Middle East respiratory syndrome (MERS) survivors developed PTSD after 6 months of being discharged from hospitals (7). Many COVID-19 survivors experience physical and mental distress symptoms such as dyspnea, acute respiratory distress (ARDS), gatism, alterations of conscious states, and traumatic medical treatments (e.g., tracheotomy) (4, 8), that could trigger PTSS and contribute to lowered quality of life (QOL), functional impairment, frequent rehospitalizations, and financial strain, and/or increased risk for depression, insomnia, and anxiety (9–14).

The presence of PTSS in COVID-19 survivors has gained research attention (4, 8, 12, 15, 16). To date, several studies have focused on PTSS in COVID-19 survivors (13, 17, 18). However, such research has had several limitations. For example, non-infected controls (controls hereafter) have not been included for comparisons and no studies, to date, have been conducted during the post-COVID-19 era, i.e., starting from May, 2020 (19, 20), in countries that have successfully controlled the epidemic. In addition, the impact of PTSS on QOL in COVID-19 survivors has been understudied, even though QOL is a widely used health outcome in clinical research and practice.

In order to reduce negative outcomes caused by PTSS in COVID-19 survivors in the post-epidemic era, it is important to understand the frequency of PTSS and its correlates within this population. Therefore, we examined the prevalence of PTSS among Chinese COVID-19 survivors during the post-epidemic era, and explored its associations with demographics, depressive symptoms, and QOL. We hypothesized that the prevalence of PTSS in COVID-19 survivors would be higher than that of controls. In addition, COVID-19 survivors with PTSS were expected to have lower overall QOL than those without.

## Methods

### Study Setting and Participants

This cross-sectional study was conducted between May 27 and September 4, 2020 at the Chongqing Mental Health Center which was responsible for follow-up assessments of mental health among all COVID-19 survivors in Chongqing Municipality. Inclusion criteria for survivors were as follows: (1) aged 18 years or above; (2) residents in Chongqing, China; (3) full recovery from COVID-19 based on a review of medical records; (4) ability to understand the contents of the assessment. Potential volunteers with major medical conditions and severe psychiatric disorders were excluded because such persons are not typically followed up in this service. COVID-19 survivors receiving regular assessments at Chongqing Mental Health Center were consecutively invited to participate in this study during the study period. Healthy controls (controls hereafter) were recruited using a convenience sampling method from the community in Chongqing. Inclusion criteria for controls were an age of 18 years or above and a status of “never infected with COVID-19”. All participants provided written informed consent forms. This study was approved by the human research ethics committee of the Chongqing Mental Health Center.

### Assessment Tools

The assessment was carried out by a research psychiatrist in a consultation room when COVID-19 survivors attended their follow-up appointments. Demographic information and clinical characteristics were assessed. PTSS was assessed using the 17-item self-report PTSD Checklist-Civilian Version (PCL-C), Chinese version (21). PCL-C items are rated on a 5-point Likert scale from 1 (“not at all”) to 5 (“extremely”) covering three domains, including intrusion (items 1-5; PCL-1), avoidance and numbing (items 6–12; PCL-2), and hyperarousal (items 13-17; PCL-3). Following previous studies (22), PCL-C total scores of ≥ 38 reflected “clinically relevant PTSS.” The Chinese version of the PCL-C has satisfactory psychometric properties (23). Severity of fatigue symptoms was assessed using a numeric rating scale (NRS) (24) scored from “0” (no suffering from fatigue) to “10” (unbearable suffering from fatigue) (25). The self-report 9-item Patient Health Questionnaire (PHQ-9), Chinese version, was used to measure severity of depressive symptoms (26–28). Each PHQ item is scored from 0 (not at all) to 3 (nearly every day). The PHQ-9 - Chinese version has satisfactory psychometric properties (29). Responses on the first two items of the World Health Organization Quality of Life-brief version (WHOQOL-BREF) were summed to provide an index of overall QOL (30–32); higher total scores indicated better QOL. Participants were also asked COVID-19 related questions, including whether their family members had been infected with COVID-19, whether they felt online mental health services were helpful, whether they experienced economic losses during the COVID-19 outbreak, and whether they frequently received news about COVID-19 through social media. Participants answered these questions with binary responses (“yes” or “no”). For COVID-19 survivors, data on severity of COVID-19 (mild/moderate vs. severe) and hospitalization due to COVID-19 were collected through a review of medical records.

### Data Analysis

Data were analyzed using the IBM Statistical Package for Social Science (SPSS) program, version 23.0. A P-P Plot was used to test normality of continuous variables. To compare demographic and clinical characteristics including PCL-1, PCL-2, PCL-3 scores between COVID-19 survivors and healthy controls, and between COVID-19 survivors with and without PTSS, Chi-square tests, independent samples *t*-tests and Mann-Whitney U Tests were used, as appropriate. Analysis of covariance (ANCOVA) was conducted to compare PTSS total score between COVID-19 survivors and healthy controls and also compare overall QOL between COVID-19 survivors with and without PTSS after controlling for variables that had statistically significant differences in univariate analyses (*P* < 0.05). Binary logistic regression analysis with the “Enter” method was conducted to examine independent demographic and clinical correlates of PTSS in the COVID-19 survivors, with PTSS as the dependent variable and other measures that had *P*-values of < 0.05 in univariate analyses as independent variables. The significance level was set at *P* < 0.05 (two-tailed).

## Results

Altogether, 138 COVID-19 survivors were invited. Of these, 134 met the study entry criteria and agreed to join this study. In addition, 214 controls were recruited during the study period. Table 1 shows differences in demographics and clinical characteristics between COVID-19 survivors and controls. All PTSS survivors were hospitalized due to COVID-19 during the pandemic.

**Table 1:** Socio-demographical and clinical characteristics of the participants.

**Variable**	**Total** **(*****N*** **=** **348)**		**Healthy controls** **(*****N*** **=** **214)**		**COVID-19 survivors** **(*****N*** **=** **134)**		**Univariate analyses**		
	***N***	**%**	***N***	**%**	***N***	**%**	**χ^**2**^**	**df**	***P***
Male gender	107	30.75	53	24.77	54	40.30	9.336	1	**0.002**
Married	232	66.67	142	66.36	90	67.16	0.024	1	0.876
Education (college and above)	225	64.66	163	76.17	62	46.27	32.236	1	** <0.001**
Living with family	267	76.72	162	75.70	105	78.36	0.326	1	0.568
Having infected family members	110	31.61	13	6.07	97	72.39	167.621	1	** <0.001**
Feel online mental service helpful	85	24.43	66	30.84	19	14.18	12.393	1	** <0.001**
Perceived economic loss	84	24.14	32	14.95	52	38.81	25.603	1	** <0.001**
Frequently receive news about COVID-19 through social media	209	60.06	142	66.36	67	50.00	9.189	1	**0.002**
Unemployed	45	12.93	14	6.54	31	23.13	20.149	1	** <0.001**
Perceived economic status	-	−	-	−	-	−	−	-	-
Poor	104	29.89	44	20.56	60	44.78	23.060	1	** <0.001**
Fair/Good	244	70.11	170	79.44	74	55.22			
Perceived health status	–	−	-	−	-	−	−	-	**-**
Poor	18	5.17	5	2.34	13	9.70	9.113	1	**0.003**
Fair/Good	330	94.83	209	97.66	121	90.30			
Having PTSS	37	10.63	12	5.61	25	18.66	14.767	1	** <0.001**
**Variable**	**Mean**	**SD**	**Mean**	**SD**	**Mean**	**SD**	***t/Z***	**df**	***P***
Age (years)	38.58	11.75	35.76	9.34	43.09	13.69	29.507	346	** <0.001**
Fatigue total	3.11	2.44	3.62	2.36	2.30	2.38	−5.393	-^a^	** <0.001**
PHQ-9 total	5.71	5.61	5.04	5.20	6.78	6.08	−2.822	-^a^	**0.005**
PCL-C total score	25.67	9.58	23.01	8.27	29.90	10.02	10.997	346	**0.001**
PCL-1	7.03	0.16	6.19	2.59	8.36	3.24	−7.979	-^a^	** <0.001**
PCL-2	10.36	0.22	8.90	3.21	12.69	4.41	−9.701	-^a^	** <0.001**
PCL-3	8.28	0.18	7.92	3.57	8.85	3.17	−3.947	-^a^	** <0.001**

*Bolded values: P < 0.05; COVID-19, Coronavirus Disease 2019; PHQ-9, the nine-item Patient Health Questionnaire; SD, standard deviation*.

a *Mann-Whitney U Test. QOL, Quality of Life; PTSS, Post-traumatic Stress Symptoms; PCL-C, the 17-item self-report PTSD Checklist-Civilian Version; PCL-1, the PCL-C items 1-5; PCL-2, the PCL-C items 6-12; PCL-3, the PCL-C items 13-17*.

Among COVID-19 survivors, the PTSS prevalence (PCL-C total scores of ≥38) was 18.66% (95%CI: 11.98–25.34%), which was significantly higher than the rate (5.61%, 95%CI: 2.50%-8.71%) found in healthy controls (*P* < 0.001). The mean PCL-C total score was higher in COVID-19 survivors (29.90 ± 10.02) than healthy controls (23.01 ± 8.27) (*P* = 0.001). Similarly, mean PCL-1 (*P* < 0.001), PCL-2 (*P* < 0.001), and PCL-3 (*P* = 0.001) scores were higher in COVID-19 survivors (Table 1). After controlling for covariates (i.e., male gender, education level, having infected family members, feeling online mental service helpful, perceived economic loss, frequently receiving news about COVID-19 through social media, unemployed, perceived economic status, perceived health status, age, and fatigue), an analysis of covariance (ANCOVA) showed that COVID-19 survivors still had a higher mean PTSS total score than healthy controls did [*F*_(1,348)_ = 4.664, *P* = 0.032].

Within the sample of COVID-19 survivors, those who reported PTSS were more likely to have family members infected with COVID-19 (*P* = 0.015), perceived economic loss (*P* = 0.001), lower perceived economic status (*P* < 0.001), poorer perceived health status (*P* = 0.016), higher total fatigue scores (*P* < 0.001), higher total PHQ-9 scores (*P* < 0.001) and lower QOL (*P* < 0.001). PCL-C total scores and PCL-1, PCL-2, and PCL-3 scores were significantly higher in COVID-19 survivors with PTSS than those without (*P* < 0.001) (Table 2). COVID-19 severity was not associated with PTSS (*P* = 0.169). After controlling for covariates using ANCOVA, there were no significant differences in overall QOL between COVID-19 survivors with and without PTSS [*F*_(1,348)_ = 1.067, *P* = 0.304).

**Table 2:** Differences between COVID-19 survivors with PTSS and those without.

**Variable**	**Total** **(*****N*** **=** **134)**		**No PTSS** **(*****N*** **=** **109)**		**PTSS** **(*****N*** **=** **25)**		**Univariate analyses**		
	***N***	**%**	***N***	**%**	***N***	**%**	**χ^**2**^**	**df**	***P***
Male gender	54	40.30	48	44.37	6	24	3.393	1	0.065
Married	90	67.16	77	70.64	13	52	3.205	1	0.073
Education (college and above)	62	46.27	54	49.54	8	32	2.517	1	0.113
Living with family	105	78.36	89	81.65	16	64	3.736	1	0.053
Having infected family members	97	72.39	74	67.89	23	92	5.914	1	**0.015**
Feel online mental service helpful	19	14.18	16	14.68	3	12	0.120	-^b^	0.729
Perceived economic loss	52	38.81	35	32.11	17	68	11.031	1	**0.001**
Use social media frequently	67	50.00	54	49.54	13	52	0.049	1	0.825
Unemployed	31	23.13	87	79.82	16	64	2.861	1	0.091
Perceived economic status	-	-	-	-	-	-	-	-	-
Poor	60	44.78	41	37.61	19	76	12.118	1	** <0.001**
Fair/Good	74	55.22	68	62.39	6	24			
Perceived health status	-	-	-	-	-	-	-	-	-
Poor	13	9.70	7	6.42	6	24	- ^b^	-^b^	**0.016**
Fair/Good	121	90.30	102	93.58	19	76			
Hospitalized due to COVID-19	-	-	-	-	-	-	-	-	-
Yes	133	99.25	108	99.08	25	100	- ^b^	- ^b^	1.000
No	1	7.46	1	0.92	0	0			
Severity of COVID-19	-	-	-	-	-	-	-	-	-
Mild/Moderate cases	126	94.03	104	95.41	22	88	- ^b^	- ^b^	0.169
Severe cases	8	5.97	5	4.59	3	12			
**Variable**	**Mean**	**SD**	**Mean**	**SD**	**Mean**	**SD**	***t/Z***	**df**	***P***
Age (years)	43.09	13.69	42.37	13.56	46.24	14.08	−1.279	132	0.203
Fatigue total	2.30	2.38	1.83	2.09	4.36	2.48	−4.646	-^a^	** <0.001**
PHQ-9 total	6.78	6.08	4.79	4.18	15.44	5.47	−6.858	-^a^	** <0.001**
Overall QOL	5.89	1.22	6.11	1.12	4.92	1.22	4.722	132	** <0.001**
PCL-C total score	29.90	10.02	26.16	5.73	46.24	8.19	−7.789	-^a^	** <0.001**
PCL-1	8.36	3.24	7.24	2.08	13.24	2.88	−7.421	-^a^	** <0.001**
PCL-2	12.69	4.41	11.14	2.74	19.48	3.89	−7.445	-^a^	** <0.001**
PCL-3	8.85	3.17	7.78	2.07	13.52	2.87	−7.416	-^a^	** <0.001**

*Bolded values: P < 0.05; COVID-19, Coronavirus Disease 2019; PHQ-9, the 9-item Patient Health Questionnaire; SD, standard deviation*.

a *Mann-Whitney U Test. QOL, Quality of Life; PCL-C, the 17-item self-report PTSD Checklist-Civilian Version; PCL-1, the PCL-C items 1-5; PCL-2, the PCL-C items 6-12; PCL-3, the PCL-C items 13-17*.

b *Fisher's Exact Test; PTSS, Post-traumatic Stress Symptoms*.

A multiple logistic regression analysis revealed that only more severe depressive symptoms (OR = 1.425, *P* < 0.001) were independently and significantly associated with higher risk of PTSS compared to its absence (Table 3).

**Table 3:** Multiple logistic regression analysis of factors independently associated with PTSS among COVID-19 survivors.

**Variables**	**Multiple logistic regression analysis**		
	***P*-value**	**OR**	**95% CI**
Having infected family members	0.083	7.744	0.765–78.377
Perceived economic loss	0.630	2.639	0.331–21.061
Perceived economic status (poor vs. fair/good)	0.950	1.069	0.133–8.607
Perceived health status (poor vs. fair/good)	0.962	0.941	0.080–11.043
Fatigue total score	0.524	1.102	0.818–1.483
PHQ-9 total score	** <0.001**	**1.425**	**1.209–1.681**

*Bolded values: P < 0.05; CI, confidential interval; OR, odds ratio; PTSS, Post-traumatic Stress Symptoms; PHQ-9, the 9-item Patient Health Questionnaire*.

## Discussion

To the best of our knowledge, this is the first study to examine PTSS in COVID-19 survivors vs. non-infected controls during the post-COVID-19 era as well as correlates of PTSS among those who had COVID-19. Compared with healthy controls, COVID-19 survivors were more likely to suffer from PTSS in the post-COVID-19 era. We found that the prevalence of PTSS (PCL-C total score of ≥38) was 18.66% in COVID-19 survivors, which is lower than corresponding figures for Italy (28%) using the PTSD Checklist for DSM-5 (PCL-5) (18) and Shenzhen, China (31%) using the Post-traumatic-stress Disorder Self-rating Scale (PTSD-SS) (17), but higher than the rate in Korea (10%) using the Impact of Event Scale-Revised Korean version (IES-R-K) (13). Reasons for discrepancies between studies could include the use of different measurement tools of PTSS, population differences in COVID-19 prevalence (e.g., Italy vs. China) and stages of the COVID-19 pandemic. However, it should be noted that previous studies of PTSD in COVID-19 survivors did not include healthy controls. Hence, the severity of PTSS in COVID-19 survivors vs. non-infected cohorts could not be elucidated in other studies.

Compared with the prevalence of PTSS in survivors of SARS (over 50%) (6) and MERS (39%) (7), the PTSS prevalence in COVID-19 survivors was substantially lower in our sample. Several possible factors could account for these discrepancies. First, mortality and complications of the three diseases caused by coronaviruses are different. Although COVID-19 is more infectious than SARS or MERS and has caused a global pandemic (33), SARS and MERS occurred earlier and were associated with higher mortality rates (34). For instance, the total mortality from COVID-19 cases is around 2% (35), while corresponding figures were 10% during the 2003 SARS outbreak and 34% during the MERS outbreak (34–36). In addition, the median intensive care unit (ICU) stay duration for SARS and MERS survivors were 15 and 11 days (37, 38), both of which are longer than that (8 days) of COVID-19 survivors (39). Moreover, the long-term complications of SARS and MERS among survivors were more persistent and severe. Longitudinal studies and systematic reviews found that SARS and MERS survivors had persistent lung function impairments at six months after discharge from ICU; most SARS survivors had extrapulmonary conditions, particularly muscle wasting and weakness (7, 40). In contrast, although fatigue and physical impairments may be common (49.6%) among COVID-19 survivors, respiratory sequelae caused by COVID-19 appear to be less severe than they are for SARS survivors (41). On these grounds, compared to SARS and MERS survivors, COVID-19 survivors generally have a better prognosis, which could be associated with less risk of PTSS. Second, based on lessons learned and experience gained from earlier viral outbreaks, timely community and psychological interventions with relevant supports have been provided during and after the COVID-19 outbreak compared to SARS and MERS outbreaks in China. Prevention efforts and intervention support have also contributed to a lower prevalence of PTSS in COVID-19 survivors (42–44).

Although the prevalence of PTSS in COVID-19 survivors was lower than rates of SARS and MERS survivors reported in the literature, its severity was still significantly higher than that of healthy controls in this study, possibly due to certain factors. First, COVID-19 patients need to undergo quarantine and receive treatments in designated hospitals. This treatment could lead to great stigma and discrimination, fear of death and spread to others as well as higher levels of stress even after full recovery (45, 46). Second, following their discharge from hospitals, COVID-19 survivors had to receive long-term regular follow-up assessments and adapt to these changes in their regular lifestyle (47). In addition, patients with chronic complications would be subjected to prolonged hospital stays, long-term maintenance treatments and rehabilitation (48, 49), and uncertainty about whether they would make a full recovery or experience future exacerbations in lingering symptoms. All these factors could magnify physical and mental distress and increase the risk for PTSS among COVID-19 survivors compared to controls (8).

In analyses of COVID-19 survivor subgroup differences, depressive symptoms were elevated among those who reported PTSS (OR = 1.425) compared to those who did not. The relationship between depressive symptoms and PTSS appears to be bidirectional (50, 51) On the one hand, depressive symptoms associated with trauma could increase the risk of PTSS (52). Conversely, distressing and debilitating symptoms of PTSD (i.e., re-experiencing and getting emotionally upset by trauma experiences) could trigger or worsen pre-existing depressive symptoms (53). In addition, there is solid evidence implicating substantial familial and genetic overlaps between PTSD and depression which may contribute to the higher risk of PTSS found among depressed patients (54). Furthermore, certain items from both the PCL-C and PHQ-9 are similar (e.g., loss of interest, suicidal ideation, sleep disturbance, and concentration difficulties) and could contribute, in part, to the association between depressive symptoms and PTSS among COVID-19 survivors.

PTSS is also associated with a range of negative outcomes such as functional impairment, and increased risk of depression, insomnia, and anxiety (9–14); presumably both PTSS and associated adverse outcomes lower QOL. Unexpectedly, in this study no significant difference in QOL between COVID-19 survivors with PTSS and without PTSS (*P* = 0.304) was found. Perhaps timely psychological interventions and social support for COVID-19 patients helped to offset adverse effects of the infection on QOL. For instance, to reduce the negative impact of COVID-19 on health outcomes of patients and their families, the Chinese government released a white paper entitled “*Fighting COVID-19: China in Action”* and stated that all treatment expenses treating COVID-19 that are not covered by basic health insurance would be reimbursed by the central budget (55). Furthermore, the National Health Commission of China (NHC) integrated psychological crisis interventions into standard treatments for COVID-19 (42). Mental health associations and academic societies such as the Chinese Psychological Society, the Chinese Society of Psychiatry, and the Chinese Association of Mental Health also published guidelines and expert consensus for people affected by the pandemic (56). All these measures could help to offset the negative impact of PTSS on QOL of COVID-19 survivors.

Strengths of this study included the addition of healthy controls to facilitate group comparisons, the focus on COVID-19 survivors in a post-COVID-19 era characterized by a low rate of new infections, and use of standardized measures on PTSS, depression, and QOL with validity support in Chinese samples. However, several methodological limitations should be acknowledged. First, because the sample size was relatively small, more sophisticated analyses such as network analysis could not be performed. Second, volunteers with major medical conditions and severe psychiatric disorders were not included in this study; given that such groups have an elevated risk for PTSD, the rate of PTSS in this sample may have been underestimated compared to the rate that may be found in the general population. Third, due to the cross-sectional design, causal relationships between PTSS and other variables could not be examined. Fourth, to mitigate response burdens on unpaid research volunteers, some factors associated with PTSS such as family history of psychiatric disorders, use of psychotropic medications, select clinical information on COVID-19 [e.g., objective test results reflecting medical complications of COVID-19, length of hospitalization and intensive care unit (ICU) treatment], were not assessed in this study. Fifth, comparisons of controls with PTSS vs. controls without PTSS were not undertaken due to the very small number of cases in the former group. Finally, for logistical reasons, only validated self-report scales [i.e., the PHQ-9 and PCL-C) were used in this study and interview-based diagnoses of psychiatric disorders, such as PTSD, could not be conducted. Structured diagnostic interview schedules (e.g., the Structured Clinical Interview for DSM (SCID)] based on face-to-face interviews should be adopted to complement questionnaire assessments, where possible, in future studies.

In conclusion, this study indicated PTSS were more severe among COVID-19 survivors compared to controls during the post-COVID-19 era. Considering the lingering experiences of emotional distress in the former group, follow-up assessments of PTSS should be conducted for COVID-19 survivors and appropriate treatments should be provided to those in need.

## Data Availability Statement

The human research ethics committee of Chongqing Mental Health Center that approved the study prohibits the authors from making the research data set publicly available. Readers and all interested researchers may contact Dr. Guo-Qing Jiang (Email address: 1159424975@qq.com) for Jiang. Dr. Li could apply to human research ethics committee of Chongqing Mental Health Center for the release of the data.

## Ethics Statement

The studies involving human participants were reviewed and approved by the human research ethics committee of the Chongqing Mental Health Center. The patients/participants provided their written informed consent to participate in this study.

## Author Contributions

YY, G-QJ, and Y-TX: study design. YY, Z-HL, Y-JZ, QZ, and LZ: data collection, analysis and interpretation. Z-HL, Y-JZ, TC, and Y-TX: drafting of the manuscript. TJ: critical revision of the manuscript. All co-authors approved the final version for publication.

## Conflict of Interest

The authors declare that the research was conducted in the absence of any commercial or financial relationships that could be construed as a potential conflict of interest.
